# Plasma carotenoids, retinol, and tocopherols and postmenopausal breast cancer risk in the Multiethnic Cohort Study: a nested case-control study

**DOI:** 10.1186/bcr2338

**Published:** 2009-07-20

**Authors:** Meira Epplein, Yurii B Shvetsov, Lynne R Wilkens, Adrian A Franke, Robert V Cooney, Loïc Le Marchand, Brian E Henderson, Laurence N Kolonel, Marc T Goodman

**Affiliations:** 1 Epidemiology Program, Cancer Research Center of Hawaii, University of Hawaii, 1236 Lauhala Street, Honolulu, HI 96813, USA; 2 Department of Preventive Medicine, Keck School of Medicine, University of Southern California, 1441 Eastlake Avenue, Los Angeles, CA 90089, USA

## Abstract

**Introduction:**

Assessments by the handful of prospective studies of the association of serum antioxidants and breast cancer risk have yielded inconsistent results. This multiethnic nested case-control study sought to examine the association of plasma carotenoids, retinol, and tocopherols with postmenopausal breast cancer risk.

**Methods:**

From the biospecimen subcohort of the Multiethnic Cohort Study, 286 incident postmenopausal breast cancer cases were matched to 535 controls on age, sex, ethnicity, study location (Hawaii or California), smoking status, date/time of collection and hours of fasting. We measured prediagnostic circulating levels of individual carotenoids, retinol, and tocopherols. Conditional logistic regression was used to compute odds ratios and 95% confidence intervals.

**Results:**

Women with breast cancer tended to have lower levels of plasma carotenoids and tocopherols than matched controls, but the differences were not large or statistically significant and the trends were not monotonic. No association was seen with retinol. A sensitivity analysis excluding cases diagnosed within 1 year after blood draw did not alter the findings.

**Conclusions:**

The lack of significant associations in this multiethnic population is consistent with previously observed results from less racially-diverse cohorts and serves as further evidence against a causal link between plasma micronutrient concentrations and postmenopausal breast cancer risk.

## Introduction

An expert panel recently concluded that an inverse association of carotenoids with breast cancer risk is possible because of their antioxidant properties [1], but methodological problems and the lack of human studies presently limit scientific judgment on the relationship [2]. Assessments of the association of dietary carotenoids and tocopherols with breast cancer risk from cohort studies have yielded inconsistent results; and only a handful of prospective studies have investigated the relation of risk to serum antioxidants, also with mixed results [3,4]. In the present nested case-control study, we tested the hypothesis that plasma concentrations of carotenoids, retinol, and tocopherols are inversely related to breast cancer risk.

## Materials and methods

The study population included participants in the biospecimen subcohort of the Multiethnic Cohort Study [5,6]. Briefly, more than 215,000 African American, Caucasian, Japanese American, Latino, and Native Hawaiian adults, aged 45 to 75 years in 1993, completed a 26-page baseline questionnaire between 1993 and 1996 in Hawaii and in Los Angeles. A biospecimen subcohort was established among Multiethnic Cohort Study participants, primarily from 2001 to 2006, including 36,458 women who completed a short telephone questionnaire and provided blood (~94% fasting) and overnight or first-morning urine.

Micronutrient analyses included prediagnostic blood from 334 postmenopausal women with breast cancer identified through linkage with Hawaii and California cancer registries through 2006. Two control individuals for each case were randomly chosen from the women in the biospecimen subcohort who were alive and free of breast cancer at the age of the case's diagnosis, and who matched the case on birth year (± 1 year), race/ethnicity, location (Hawaii or California), date (± 6 months) and time (± 2 hours) of blood draw, fasting hours (8 to < 10 hours and 10+ hours), and hormone therapy use (current versus not current). Plasma concentrations of carotenoids, retinoids, and tocopherols were determined by high-pressure liquid chromatography with photodiode array detection from our earlier protocol [7,8], with slight modifications [6]. The median time from blood draw to date of diagnosis was 1 year and 5 months, with the middle 50% of subjects having follow-up time in the range of 8 months to 2.5 years.

The Multiethnic Cohort Study and the nested case-control study from the biospecimen subcohort were approved by the Institutional Review Boards of the University of Hawaii and the University of Southern California. Cohort participants provided informed consent for both studies.

Conditional logistic regression, with matched sets as strata, was used to compute odds ratios and 95% confidence intervals. Biomarker variables were categorized into quartiles based on the distribution of cases and controls in order to optimize the distribution of cases and controls in each of the strata of interest so that none of the cells had overly small numbers of subjects. A trend variable for each biomarker was created by assigning the median value of the appropriate category. Aside from matching variables, the full models were adjusted for the following established breast cancer risk factors [9]: body mass index, alcohol use, age at first birth, number of full-term pregnancies, age at menarche, and age at menopause. Interactions with smoking status, race/ethnicity, and body mass index were assessed, and a separate model was considered excluding subjects who were diagnosed within 1 year after blood draw. All analyses were conducted using SAS software (version 9.1; SAS Institute, Cary, NC, USA).

## Results

Only matched sets that included one case and at least one control with no missing data on plasma micronutrients or any of the adjustment variables were retained in these analyses, resulting in a study population of 286 cases and 535 controls. Cases were similar to controls for most baseline characteristics, except that cases were more likely to be former or current smokers and ever drinkers than controls (Table 1).

**Table 1:** Characteristics of breast cancer cases and controls

General characteristic	Cases (n = 286)	Controls (n = 535)
Age at blood draw (years)^a^	66.0 (8.1)	66.0 (8.0)
Fasting hours prior to blood draw^a^	13.1 (1.9)	13.0 (1.8)
Current hormone therapy use^a^	99 (35%)	185 (35%)
Ethnicity^a^		
African American	35 (12%)	65 (12%)
Caucasian	59 (21%)	111 (21%)
Japanese American	116 (40%)	213 (40%)
Latina	45 (16%)	86 (16%)
Native Hawaiian	31 (11%)	60 (11%)
High school education or less	97 (34%)	189 (35%)
Body mass index (kg/m^2^)	25.6 (4.9)	25.4 (5.4)
Nulliparous	35 (12%)	58 (11%)
Number of full-term pregnancies	2.7 (1.8)	2.8 (1.8)
Age at first childbirth	23.8 (4.6)	23.5 (4.5)
Age at menarche	13.0 (1.6)	13.1 (1.6)
Age at natural menopause	49.1 (5.1)	49.5 (4.7)
Smoking status		
Never	159 (56%)	334 (63%)
Former	90 (32%)	146 (27%)
Current	35 (12%)	52 (10%)
Pack-years of smoking	6.2 (11.2)	5.4 (11.0)
Mother or sister with breast cancer	31 (11%)	68 (13%)
Alcohol use		
Never	170 (60%)	347 (65%)
Ever, below users' median (< 3.4 g/day)	58 (20%)	95 (18%)
Ever, above users' median (> 3.4 g/day)	58 (20%)	93 (17%)
Hours in moderate or vigorous physical activity per day	1.2 (1.3)	1.2 (1.3)

Data presented as mean ± standard deviation or *n *(%). ^a^Matching variable.

Women with breast cancer tended to have lower levels of plasma carotenoids and tocopherols than matched controls, but the differences were not large or statistically significant and the trends were not monotonic (Table 2). When stratified by smoking status, ever smokers, but not never smokers, in the highest quartile compared with the lowest quartile of total carotenoid levels had a significantly decreased risk of breast cancer (odds ratio = 0.29, 95% confidence interval = 0.10 to 0.85 for ever smokers; odds ratio = 1.19, 95% confidence interval = 0.62 to 2.27 for never smokers). No evidence was found, however, for an interaction (*P*_interaction _= 0.17) between smoking and circulating carotenoids on risk; nor was there evidence that other variables modified the micronutrient-breast cancer risk association. A sensitivity analysis excluding cases diagnosed within 1 year after blood draw did not alter the findings.

**Table 2:** Risk of breast cancer across quartiles of plasma carotenoids, tocopherols, and retinol^a^

Antioxidant (ng/ml)	Number of cases/controls^b^	Odds ratio^c^	95% confidence interval
Total carotenoids			
≤ 1,129.4	76/129	1.00	
1,129.5 to 1,510.2	71/135	0.88	0.58 to 1.32
1,510.3 to 1,953.0	73/132	0.90	0.59 to 1.37
≥ 1,953.1	66/139	0.80	0.51 to 1.26
		(*P*_trend _= 0.39)	
Total carotenes			
≤ 231.7	79/126	1.00	
231.8 to 373.5	66/139	0.75	0.49 to 1.14
373.6 to 569.5	82/123	0.99	0.64 to 1.52
≥ 569.6	59/147	0.61	0.38 to 0.97
		(*P*_trend _= 0.08)	
α-Carotene			
≤ 42.0	73/132	1.00	
42.1 to 67.5	70/135	0.95	0.62 to 1.45
67.6 to 101.7	75/131	1.06	0.69 to 1.64
≥ 101.8	68/137	0.88	0.56 to 1.39
		(*P*_trend _= 0.64)	
β-Carotene			
≤ 180.4	79/127	1.00	
180.5 to 301.9	64/140	0.74	0.49 to 1.13
302.0 to 460.8	79/126	0.97	0.63 to 1.48
≥ 460.9	64/142	0.73	0.46 to 1.15
		(*P*_trend _= 0.30)	
*trans*-β-Carotene			
≤ 168.8	79/126	1.00	
168.9 to 283.7	65/140	0.76	0.50 to 1.14
283.8 to 432.5	76/129	0.91	0.59 to 1.40
≥ 432.6	66/140	0.76	0.49 to 1.19
		(*P*_trend _= 0.37)	
*cis*-β-Carotene			
≤ 11.9	78/128	1.00	
12.0 to 19.5	72/133	0.89	0.59 to 1.33
19.6 to 30.5	75/129	0.91	0.60 to 1.38
≥ 30.6	61/145	0.68	0.43 to 1.07
		(*P*_trend _= 0.10)	
Lycopene			
≤ 218.7	73/133	1.00	
218.8 to 292.2	75/130	1.04	0.68 to 1.58
292.3 to 391.7	70/135	0.91	0.06 to 1.37
≥ 391.8	68/137	0.88	0.57 to 1.38
		(*P*_trend _= 0.50)	
*trans*-Lutein/zeaxanthin			
≤ 186.8	77/129	1.00	
186.9 to 246.5	70/135	0.92	0.60 to 1.41
246.6 to 312.5	70/135	0.91	0.59 to 1.41
≥ 312.6	69/136	0.87	0.57 to 1.34
		(*P*_trend _= 0.55)	
*trans*-Lutein			
≤ 129.7	79/126	1.00	
129.8 to 174.4	63/143	0.74	0.48 to 1.12
174.5 to 223.4	74/131	0.94	0.62 to 1.44
≥ 223.5	70/135	0.85	0.55 to 1.29
		(*P*_trend _= 0.68)	
*trans*-Zeaxanthin			
≤ 53.6	71/134	1.00	
53.7 to 70.7	80/126	1.17	0.78 to 1.77
70.8 to 92.2	69/135	0.95	0.60 to 1.50
≥ 92.3	66/140	0.88	0.56 to 1.39
		(*P*_trend _= 0.44)	
*cis*-Lutein/zeaxanthin			
≤ 90.8	79/127	1.00	
90.9 to 111.7	63/142	0.70	0.46 to 1.06
111.8 to 137.8	77/128	0.90	0.59 to 1.37
≥ 137.9	67/138	0.75	0.48 to 1.16
		(*P*_trend _= 0.37)	
*trans*-Anhydrolutein			
≤ 40.0	76/129	1.00	
40.1 to 54.0	71/135	0.88	0.57 to 1.36
54.1 to 73.6	74/131	0.94	0.62 to 1.43
≥ 73.7	65/140	0.78	0.50 to 1.22
		(*P*_trend _= 0.31)	
*cis*-Anhydrolutein			
≤ 32.9	67/138	1.00	
33.0 to 44.5	73/132	1.20	0.78 to 1.84
44.6 to 57.7	73/133	1.21	0.78 to 1.87
≥ 57.8	73/132	1.21	0.77 to 1.91
		(*P*_trend _= 0.47)	
α-Cryptoxanthin			
≤ 30.8	67/138	1.00	
30.9 to 38.9	79/126	1.28	0.84 to 1.96
39.0 to 49.5	71/135	1.10	0.72 to 1.68
≥ 49.6	69/136	1.08	0.68 to 1.70
		(*P*_trend _= 0.96)	
*trans*-β-Cryptoxanthin			
≤ 93.8	69/137	1.00	
93.9 to 169.0	77/127	1.22	0.81 to 1.85
169.1 to 293.7	61/145	0.85	0.55 to 1.33
≥ 293.8	79/126	1.36	0.85 to 2.16
		(*P*_trend _= 0.30)	
*cis*-β-Cryptoxanthin			
≤ 36.4	69/136	1.00	
36.5 to 53.9	72/133	1.08	0.71 to 1.64
54.0 to 87.0	67/139	0.96	0.62 to 1.50
≥ 87.1	78/127	1.27	0.81 to 1.99
		(*P*_trend _= 0.28)	
Retinol			
≤ 842.6	66/139	1.00	
842.7 to 997.9	76/130	1.29	0.85 to 1.95
998.0 to 1,187.9	72/133	1.18	0.75 to 1.86
≥ 1,188.0	72/133	1.13	0.73 to 1.76
		(*P*_trend _= 0.73)	
Total tocopherols			
≤ 14,413.3	78/128	1.00	
14,413.4 to 17,909.5	73/132	0.95	0.63 to 1.42
17,909.6 to 23,282.3	63/142	0.74	0.48 to 1.14
≥ 23,282.4	72/133	0.86	0.56 to 1.31
		(*P*_trend _= 0.44)	
α-Tocopherol			
≤ 11,828.1	80/125	1.00	
11,828.2 to 16,021.0	68/138	0.79	0.52 to 1.20
16,021.1 to 21,829.8	66/138	0.75	0.49 to 1.16
≥ 21,829.9	72/134	0.80	0.52 to 1.24
		(*P*_trend _= 0.42)	
β + γ-Tocopherol			
≤ 619.0	64/142	1.00	
619.1 to 1,243.2	71/134	1.24	0.80 to 1.91
1,243.3 to 2,175.2	76/129	1.32	0.87 to 2.02
≥ 2,175.3	75/130	1.40	0.86 to 2.27
		(*P*_trend _= 0.22)	
δ-Tocopherol			
≤ 190.1	71/134	1.00	
190.2 to 257.1	60/145	0.78	0.48 to 1.25
257.2 to 339.2	85/121	1.35	0.82 to 2.22
≥ 339.3	70/135	0.99	0.58 to 1.70
		(*P*_trend _= 0.57)	

^a^Linear dose–response in the logit of risk was tested by a Wald test for each biomarker modeled as a trend variable assigned the median value of the appropriate category. ^b^Controls (n = 535) were women matched to cases (n = 286) on geographic area, ethnicity, year of birth, date and time of specimen collection, fasting status, and hormone therapy use. ^c^Adjusted by conditional logistic regression with matched sets as strata, with additional adjustment for age at blood draw and fasting hours prior to blood draw (as continuous variables), as well as body mass index, alcohol use, age at menarche, age at menopause, age at first birth, and number of full-term pregnancies.

## Discussion

The present case-control study – nested within a large prospective cohort – does not support an association of plasma carotenoids, retinol, or tocopherols with postmenopausal breast cancer risk.

The observation of a lack of an association between breast cancer risk and plasma tocopherols and retinol mirrors what has been seen in recent prospective studies [4,10-13]. Additionally, a recent clinical trial did not observe any change in risk of breast cancer among women assigned to receive vitamin E supplements [14]. The carotenoid findings, too, are consistent with the null results from the Shanghai Women's Health Study [4]. Five other recent large (> 100 cases) prospective studies of plasma antioxidants and risk of breast cancer, however, did report some significant associations with carotenoids. Four of the five studies found breast cancer risk to be inversely associated with α-carotene, β-carotene, and/or total carotenoids [11-13,15]; two of the five studies observed an inverse association with lycopene [10,13], and one of these also observed an inverse association with γ-tocopherol [13].

The suggested inverse association of carotenoids with breast cancer risk limited to ever smokers, observed in our study, is biologically plausible as cigarette smoke contributes to oxidative stress and a reduction in plasma carotenoids [16]. Accordingly, smokers stand to benefit more from the antioxidant properties of carotenoids. Among the two previous studies that examined the breast cancer-circulating carotenoid association by smoking status, however, one reported that the inverse association of α-carotene with breast cancer risk may be limited to never smokers and past smokers (*P*_interaction _= 0.10) [12]; the other study found no evidence for a smoking-circulating carotenoid interaction on breast cancer risk after restricting the analysis to women who were diagnosed with breast cancer at least 2 years after blood draw [10]. Two recent studies of dietary antioxidants and breast cancer risk from the Collaborative Breast Cancer Study and the Nurses' Health Study, however, found decreases in risk with increases in carotenoids among current smokers only, similar to the present study [17,18].

Limitations to the current study include the use of only one measure to assess exposure, the possibility that the exposure was not assessed during the relevant time period, a relatively short follow-up time, and the inability to stratify by tumor characteristics. These limitations would most probably bias the risk estimates toward the null. We were also unable to assess whether the associations we observed with carotenoids were due to factors other than a high consumption of fruits and vegetables that are also related to a healthy lifestyle. We did, however, adjust for maximum years of education obtained, and saw no significant changes in the results. The study is strengthened by its multiethnic population, which had similarly high levels of antioxidants, including carotenoids, as those reported in the Nurses' Health Study [12]. These levels are higher than those reported in the majority of the previous studies [10,11,13,15]. The difference in antioxidant levels could, however, be due to laboratory variability and/or due to the diet of our population.

## Conclusions

The lack of significant associations in the present multiethnic population is consistent with previously observed results from less racially-diverse cohorts and serves as further evidence against a causal link between plasma micronutrient concentrations and postmenopausal breast cancer risk.

## Competing interests

The authors declare that they have no competing interests.

## Authors' contributions

All of the authors made substantial contributions to the study concept and design or analysis and interpretation of the data. Specifically, ME designed the analysis, analyzed the results, and was the primary author of every section of the text. YBS aided in the design of the analysis and performed much of the initial statistical analyses. LRW helped to design the study's analytic strategy and prepare the Materials and methods section of the text. AAF and RVC carried out the micronutrient assays. LLM, BEH, and LNK were instrumental in the design of the study, and commented on and approved the manuscript. MTG originally conceived of the study and helped to draft the manuscript.
